# Practical hardware for evolvable robots

**DOI:** 10.3389/frobt.2023.1206055

**Published:** 2023-08-21

**Authors:** Mike Bilby, Edgar Buchanan, Léni K. Le Goff, Emma Hart, Agoston E. Eiben, Matteo De Carlo, Alan F. Winfield, Matthew F. Hale, Robert Woolley, Jon Timmis, Andy M. Tyrrell

**Affiliations:** ^1^ School of Physics, Engineering and Technology, University of York, York, United Kingdom; ^2^ School of Computing, Edinburgh Napier University, Edinburgh, United Kingdom; ^3^ Department of Computer Science, Vrije Universiteit Amsterdam, Amsterdam, Netherlands; ^4^ Bristol Robotics Laboratory, University of the West of England, Bristol, United Kingdom; ^5^ School of Computer Science, University of Sunderland, Sunderland, United Kingdom

**Keywords:** evolutionary robotics, hardware design, modular robots, hardware constraints, autonomous robot fabrication, robot manufacturability

## Abstract

The evolutionary robotics field offers the possibility of autonomously generating robots that are adapted to desired tasks by iteratively optimising across successive generations of robots with varying configurations until a high-performing candidate is found. The prohibitive time and cost of actually building this many robots means that most evolutionary robotics work is conducted in simulation, but to apply evolved robots to real-world problems, they must be implemented in hardware, which brings new challenges. This paper explores in detail the design of an example system for realising diverse evolved robot bodies, and specifically how this interacts with the evolutionary process. We discover that every aspect of the hardware implementation introduces constraints that change the evolutionary space, and exploring this interplay between hardware constraints and evolution is the key contribution of this paper. In simulation, any robot that can be defined by a suitable genetic representation can be implemented and evaluated, but in hardware, real-world limitations like manufacturing/assembly constraints and electrical power delivery mean that many of these robots cannot be built, or will malfunction in operation. This presents the novel challenge of how to constrain an evolutionary process within the space of evolvable phenotypes to only those regions that are practically feasible: the viable phenotype space. Methods of phenotype filtering and repair were introduced to address this, and found to degrade the diversity of the robot population and impede traversal of the exploration space. Furthermore, the degrees of freedom permitted by the hardware constraints were found to be poorly matched to the types of morphological variation that would be the most useful in the target environment. Consequently, the ability of the evolutionary process to generate robots with effective adaptations was greatly reduced. The conclusions from this are twofold. 1) Designing a hardware platform for evolving robots requires different thinking, in which all design decisions should be made with reference to their impact on the viable phenotype space. 2) It is insufficient to just evolve robots in simulation without detailed consideration of how they will be implemented in hardware, because the hardware constraints have a profound impact on the evolutionary space.

## 1 Introduction

The objective of evolutionary robotics is to apply principles of biological evolution to artificial systems, either to generate novel designs for practical applications, or to study biological evolution itself. This can be applied at the controller level, where the robot hardware is predefined and only its behaviours are evolved, or at the morphological level, where the body plan of the robot itself is evolved. To gain the full benefit of the evolutionary approach, it is desirable to do both, but morphological evolution is very challenging to implement with real robot hardware, since it requires a system capable of realising a wide range of body plans with highly variable requirements. For this reason, evolution of real robots is often only applied at the controller level, with few examples of morphological evolution progressing beyond simulated models (Pollack and Lipson, 2000; Hiller and Lipson, 2011; Brodbeck et al., 2015; Jelisavcic et al., 2017; Auerbach et al., 2018; Kriegman et al., 2020a; Kriegman et al., 2020b). However, to achieve the objective of evolving robot bodies that are of practical use in real-world applications, they must be implemented in hardware (Eiben, 2014).

The Autonomous Robot Evolution (ARE) project^1^ sought to achieve this with an autonomous fabrication system, using a combination of 3D printing and modular functional parts to enable a wide range of evolved body plans to be rapidly implemented in hardware. This semi-modular approach is distinct from the discrete modular approach, whereby robots are constructed entirely from prefabricated modules (Brodbeck et al., 2015; Miras et al., 2020; Moreno and Faiña, 2021), and little research has been carried out with hardware robots that can take more arbitrary shapes (Samuelsen and Glette, 2015; Kriegman et al., 2020b).

The challenges of developing this complete evolutionary system were numerous and varied, and many of these are detailed in other publications, such as autonomous manufacture (Hale et al., 2019; Hale et al., 2020), evolutionary approaches (Buchanan et al., 2020a; Buchanan et al., 2020b) and robot learning (Le Goff et al., 2022). In this paper, the focus is on the challenges of developing robot hardware for implementing morphological evolution, and in particular how the design of this hardware interacts with the evolutionary process.

We have found that the practical constraints introduced by real hardware have a profound impact on the nature of the problem to be solved by the evolutionary process, and reached the conclusion that both the hardware and algorithm designers of any practical robot evolution system need to keep careful consideration of these interactions at the heart of their design decisions if the system is to be effective (Buchanan et al., 2020b).

To clarify the basis of this discussion, it is helpful to visualise the task that the evolutionary process is trying to solve. Robot evolution may be regarded as an environment-driven, population-based optimisation algorithm, whereby many different robot configurations are evaluated in a target environment and assigned a “fitness” based on their effectiveness in performing the desired task. The objective is to efficiently find the best possible configuration, i.e., the robot with the highest fitness.

This task can be visualised as exploring a vast multidimensional space comprising all the possible robot body plans that could be represented by different combinations of genes, trying to find the best one. This great crowd of potential robots is the *phenotype space*. Overlaid on this space is the *fitness landscape*, a rugged terrain made up of the fitness scores for each individual in the phenotype space. Evolution attempts to find the best phenotype by sampling and traversing this fitness landscape: evaluating generations of robots, selecting for those with higher fitness, and applying mutation and crossover operators to their corresponding genotypes with the aim of discovering ever higher “peaks” in the fitness landscape that correspond to more successful robot phenotypes.

The size of the phenotype space is very large for all but the simplest structures, and even when using evolution to explore it more efficiently, physically building and evaluating this many robots is usually prohibitive in cost and time. For this reason, much of evolutionary robotics takes place in simulation, with only selected high-fitness individuals being implemented in hardware. A known shortcoming of this approach is that the simulator does not perfectly replicate reality, meaning that simulated robots behave differently to their real counterparts, and therefore the fitness expected from their evaluation in simulation may differ from the actual fitness observed in real life. In other words, there is a discrepancy between the fitness landscape in simulation, and the fitness landscape in hardware. This discrepancy is known as the *reality gap* (Jakobi et al., 1995), and it means that the optimum phenotypes obtained by an evolutionary process operating on the fitness landscape in simulation may not actually perform well in reality.

However, there is a second, less-discussed issue with evolving robots for hardware implementation. In the simulated environment, there are very few constraints on the robots that can be built and evaluated. Energy is limitless, parts can be of arbitrary size and shape with no internal workings, and the robots can be conjured into existence without any manufacturing or assembly processes. Real hardware implementation, by contrast, imposes many practical constraints. Power is limited, and a robot that overloads its power system may become inoperable. The robot parts must contain electronic and mechanical elements to provide their functionality and connect them together, the design of which involves many compromises between competing structural, functional and financial objectives. The robots must also be physically manufactured and assembled, and every fabrication process has limitations on the shape and scale of structures that it can produce. What does all this mean for the evolutionary process?

**FIGURE 1:**
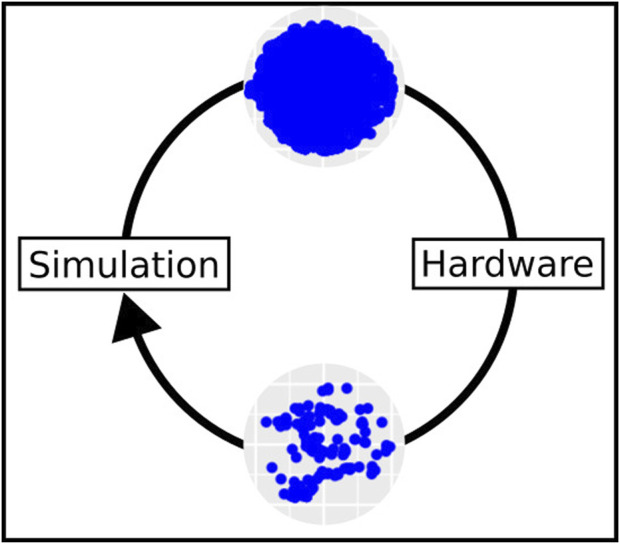
The simulation domain provides a large space of possible robots that could be evolved, represented here as blue dots (top). However, only portions of this space contain robots that are practically feasible in hardware (bottom). The scatter plots representing the two landscapes are taken from experimental data presented in Buchanan et al. (2020a).

Applying this to the phenotype space of all possible robots, it becomes apparent that many of these phenotypes are not feasible, because the constraints of real hardware mean that they cannot be successfully implemented as functional robots. This may be because they cannot be physically constructed, or because their body plans exceed the capabilities of the underlying hardware to support their operation. In effect, these unfeasible phenotypes introduce no-go regions in the phenotype space, which changes the nature of the task faced by the evolutionary process. The *evolvable phenotype space*, defined by all the possible robots that could be represented by different combinations of the evolved parameters, is a contiguous landscape in which the evolutionary algorithm can move freely, in the sense that each genotype corresponds to a valid robot that can be evaluated. The *viable phenotype space*, by contrast, is only a distributed subset of these possibilities, where regions of valid robots are broken up by unfeasible regions, and the evolutionary algorithm must somehow navigate around these obstacles in order to explore the feasible regions of the space. The difference between these two spaces is visualised in Figure 1. In this paper, we define these terms as follows:

*“The* ***evolvable phenotype space*** *is defined as the complete set of possible phenotypes that could be generated by an evolutionary process within a particular genetic representation”*

*“The* ***viable phenotype space*** *is defined as the subset of evolvable phenotypes that can be implemented and reliably evaluated in hardware, after manufacturing constraints and hardware limitations are taken into account”*

The distinction between this issue and the reality gap may be further clarified by classifying the evolutionary search space into two domains: the behavioural domain and the phenotype domain. The reality gap refers to the difference between the evolvable behavioural space and the real behavioural space, whereby imperfect simulation of reality leads to divergence in behaviour between simulated and physical robots. In principle, these are all differences that could be reduced by improving the fidelity of the simulator. By contrast, the differences between the *evolvable phenotype space* and *viable phenotype space* arise not as a result of imperfect simulation, but rather from the presence of physical hardware constraints that render certain evolved robot configurations unfeasible. This is then no longer a difference in their behaviour, but a difference between whether or not those robots can be implemented and evaluated at all.

This has two important consequences. The first is that some method is required to restrict the evolutionary process to the feasible regions of the space, and this will have an effect on its performance. An evolutionary process which is effective at exploring the *evolvable phenotype space* may not perform so well in the *viable phenotype space*. The second consequence is that the design of the hardware implementation will to a large extent define the *viable phenotype space*. That is to say, each decision taken at the hardware design level has the potential to directly influence where the valid regions of the phenotype space are, how difficult it is for evolution to move between them, and the usefulness of robots within those feasible regions to the target application. This interplay between hardware design constraints and evolution has not been discussed in detail in the literature, and exploring this is the primary objective of this paper.

The core conclusion of the paper is that consideration of the *viable phenotype space* is central to the design of any effective system for evolving practical robots, and this is relevant to both the hardware designer and the evolutionary designer. On the engineering side, making design decisions without reference to the *viable phenotype space* may result in a hardware platform that is undesirably restrictive to the evolutionary process. On the evolutionary side, the challenge of restricting evolution to feasible regions means it is insufficient just to apply algorithms that have been optimised in simulation to perform well in the *evolvable phenotype space*, because these may not be effective when required to operate within realistic practical constraints.

These pitfalls on both engineering and evolutionary sides were extensively encountered in the ARE project, as it sought to evolve both morphology and behaviour of practical robots to be implemented in hardware. This makes it an insightful case study on the interplay between hardware and evolution, and this paper will present a detailed exploration of the hardware constraints in this system and their effect on the *viable phenotype space*. It should be noted, however, that this is not a presentation of a successful system for others to replicate, but rather a reflective discussion of lessons learned through the attempt to create such a system without the benefits of the insights presented here. In doing so, the aim is to identify useful design principles for future work, and highlight areas which are commonly overlooked.

Although the hardware under discussion is specific to the ARE system, the principles are applicable to any system attempting to evolve practical robots. Even the best hardware has limitations, and pragmatic constraints such as funding and time will invariably impose significantly more severe limitations on the type and quality of hardware that can be used. The challenge, therefore, is how to shape those constraints in such a way as to make the most of the available resources, i.e., to maximise the usefulness of the *viable phenotype space*.

The remainder of this paper explores these challenges using the ARE hardware design as an illustrative case study, and will be structured as follows:

• Section 2 focuses on the engineering aspects of the hardware design, covering the key challenges of building evolved bodies, making mechanical/electrical interconnections, and the underpinning electronic hardware.
• Section 3 explores how these design decisions result in constraints on the evolutionary space, with examples of how these constraints can affect the evolutionary process.
• Section 4 draws the engineering and evolutionary aspects together to reflect on the practical implications of the previous observations, and what they mean for future work.

## 2 Materials and methods

### 2.1 Overview

**FIGURE 2:**
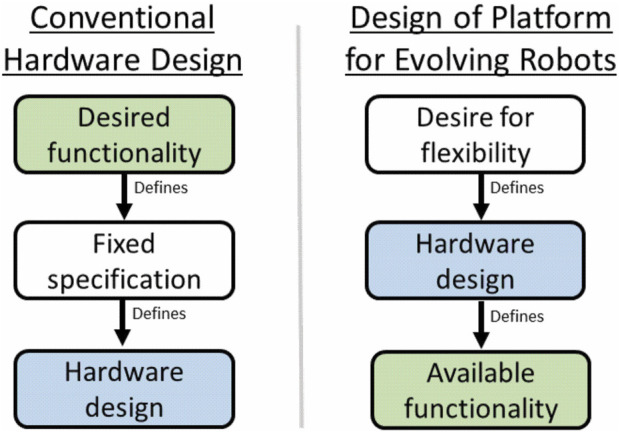
An illustration of how the hardware design paradigm changes for an evolvable robot platform. Rather than the design being defined by fixed constraints derived from the desired functionality for the robot, the hardware design comes first, and defines the constraints on the functionality available to the evolved robots.

Practical hardware design for evolvable robots presents an unusual challenge, as visualised in Figure 2. Hardware is usually designed to a fixed specification, where the requirements of the system are known ahead of time and can be used as constraints, guiding design decisions to ensure the specification is met. For an evolvable robot system capable of implementing arbitrary body plans, the specification is variable by definition, so there are very few fixed constraints on the design. In practice, this means that *the hardware design decisions will determine the constraints of the system*, and not the other way round. Designing a hardware platform for evolving robots means designing for flexibility, aiming to ensure reliable and consistent performance whilst equipping evolution with the largest possible space of potential robot body plans to explore.

The remainder of this section will begin by briefly describing the ARE framework to provide an initial understanding of the robot production system, before covering the different areas of the implementation in more specific detail in terms of the design choices and their rationale.

The robots are constructed in a semi-modular fashion, with prefabricated sensor and actuator modules known as “organs” being affixed onto a 3D printed free-form plastic “skeleton.” Inserted into the centre of each skeleton is a core unit known as the “head organ,” which provides central processing and distributes power and communications to the organs via connecting cables.

**FIGURE 3:**
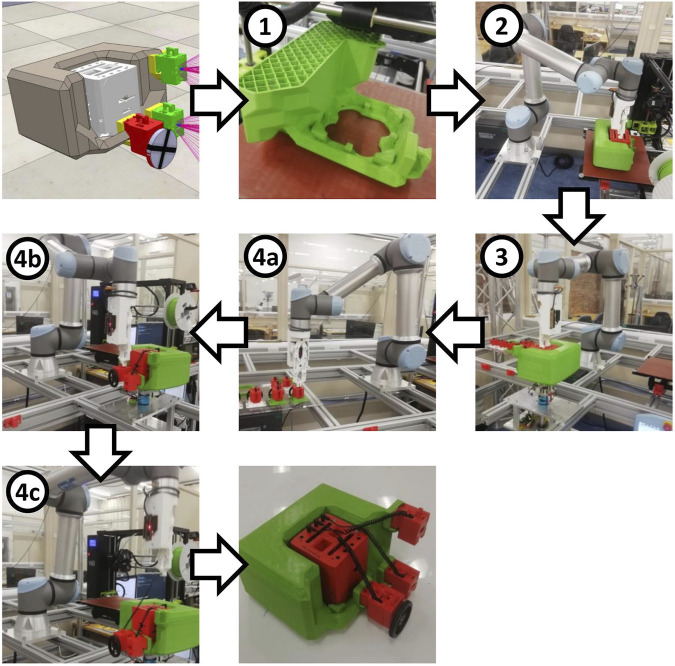
The production process to create a robot designed by evolution, from digital to physical. The step numbers correspond to those described in detail in the main text. Firstly the skeleton must be 3D printed before the head (containing the controller and battery) is inserted. Then wheels and sensors can be attached and connected to the head via retracting cables. The complete process of producing a physical robot from its digital specification is done autonomously.

These robots are created by an autonomous production process^2^ as illustrated in Figure 3. To create a new robot, a body plan is first evolved in simulation. This determines the morphology of the skeleton and the placement of organs upon it to form a complete robot. The skeleton is 3D printed 1), and then the complete robot design is autonomously assembled by a robot arm (2–4c). To assemble it, the robot arm first inserts the head organ into the skeleton 2), which is still stuck to the build plate of the 3D printer. The head organ is secured in place by a sprung clip mechanism in its base, mating with corresponding structures that have been integrated directly into the plastic of the skeleton when it was printed. The head organ then provides a secure grasping point for the robot arm to pull the printed skeleton off the build plate of the printer and place it onto an assembly stand 3). The peripheral organs have their own sprung clip mechanisms in their casings, allowing the robot arm to attach each of them to matching mating points printed into the skeleton. The connecting cable for each organ is stored within an onboard cavity, from which it can be drawn out by the robot arm and connected to the head organ to complete the assembly (4a–4c). The robot then wirelessly receives its control algorithm and is ready for operation. Additional details about the assembly process may be found in Hale et al. (2020).

A core aim in the development of the ARE system was full autonomy, i.e., the ability to manufacture evolved robots from genome to hardware phenotype without human intervention. The goal of this was to reduce some of the practical barriers to conducting evolution with real robots, which is otherwise a highly labour-intensive process. The additional requirements of this objective heavily restricted the choices of hardware for the robot platform, as will become apparent throughout the following description of the system. This highlights an important reality of designing any practical robot evolution system: it is rarely an option to simply select high-performance hardware that is the least restrictive to the evolutionary process. Application-specific design objectives such as this one, in combination with other practical, technical, and financial constraints, will invariably result in a setup that is less than optimal. The ARE system, being limited in many such ways, therefore provides insights which are of relevance to all future work, even though other implementations will differ in the exact nature of their limitations.

The upcoming subsections will consider the three key problems that must be solved by any hardware platform for evolving robots: building evolved bodies, making mechanical/electrical interconnections, and the design of underpinning electronic hardware.

### 2.2 Building evolved bodies

To manufacture generations of robots with evolved body plans, the chosen implementation is required to provide as much scope for body plan variation as possible, whilst keeping the dimensionality of the evolutionary search space small enough to make the problem tractable within an acceptable time frame. It is also desirable for the system to have a high throughput and require minimal human involvement, in order to maximise the number of robots that can be produced. The ARE system uses 3D printing to manufacture “skeletons” that enable a wide range of morphological variations to be evolved, and equips them with functional robot parts by using prefabricated modular “organs.”

#### 2.2.1 3D printed skeletons

The evolved skeletons are produced by using Fused Deposition Modeling (FDM) 3D printing to manufacture algorithmically generated Standard Triangle Language (STL) models. The objective of using this technology was to enable a very rich morphological space since the skeletons could in principle take any arbitrary form that could be manufactured by the printer. It turns out that there are a great many caveats to this assumption as discussed later, but this technology does lend itself well to realising novel morphological structures.

The Lulzbot TAZ 6^3^ printer model was selected since these printers have a large build volume, an open-framed style for easy access by the robot arm, and open-source hardware and software for easier integration into the automated ARE system. To improve throughput and model strength, the “MOARstruder” extrusion head is used, which has an oversize 1.2 mm nozzle, enabling rapid printing using a layer height of 0.9 mm. Polylactide (PLA) plastic was selected as a cheap and reliable build material.

#### 2.2.2 Modular organs

To evolve robots that are capable of intelligent interaction with the environment, arbitrary configurations of both sensors and actuators need to be combined with the body structure. The ARE approach to this is to use prefabricated modules called “organs.” These integrate all the supporting electronics and mechanical/electrical connections required for each sensor or actuator to function as part of a robot, which can then be simply combined with the 3D printed skeleton to produce a fully-functioning robot.

**FIGURE 4:**
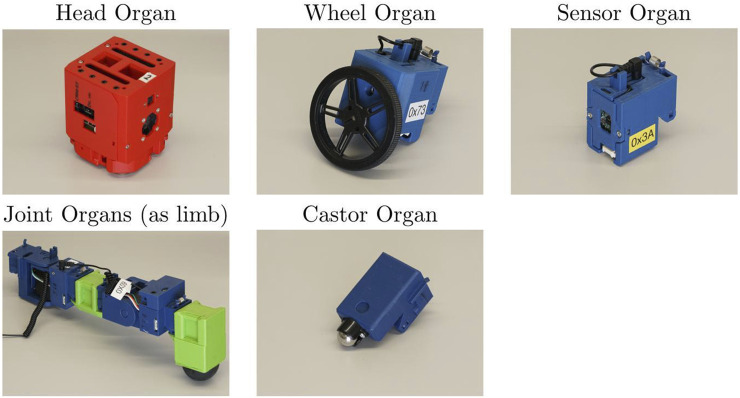
The organs available for the ARE system are the following: head, wheel, sensor, joint and castor. The controller of the robot is run in the head organ, which also supplies the other organs with power and communications. The wheel organ provides rotary locomotion. The sensor organ contains two sensors, enabling the measurement of distance from objects and the detection of infrared light. The joint organs provide powered articulation points for forming limbs, as in the pictured example of a two-jointed leg (joints in blue). The castor ball is the unique passive organ designed to reduce the friction between the robot and the floor.

Each robot is built around a central processing and power unit known as the “head organ,” which handles power distribution and communication with a variable number of peripheral organs. These are the sensor organ, wheel organ and joint organ, the latter of which can be used individually or daisy-chained to form limbs. The leg organ is a two-jointed construct of these combined with a rubber-tipped foot. There is also an additional passive organ with no electronics, the castor organ, which is a simple ball castor enabling evolution to generate free-rolling points on a robot. The organ types and their functionality are summarised in Figure 4.

### 2.3 Interconnections

Having determined how to implement the fundamental building blocks of a robot, the question of how they can be connected together to build a working robot must be considered. This is trivial in simulation, where multiple bodies can be simply instructed to stick together, communication happens through omniscience, and energy can be created at will wherever it is needed. In the physical world, however, this is not so straightforward—mechanical connections to assemble the structural parts together are required, and all active parts of the robot must be supplied with power and communication links to the controller. The choice of interconnection method will have far-reaching impacts on the performance, flexibility and ease of use of the evolvable platform.

In the ARE system, a combination of mechanical clips and headphone cables were used. Although somewhat bulky and restrictive, this is one of the simplest ways to create secure, reversible mechanical and electrical connections in this context. For example, magnetic connectors like those used in the EMERGE modular platform, Moreno and Faiña (2021) or latching PCBs such as those used in Faina et al. (2015) would not be suitable for autonomously connecting organs to a printed robot skeleton, because one-half of the connection must come straight from the 3D printer, and hence be plastic-only. This is a trade-off of having the morphological complexity afforded by 3D printing, combined with the needs of automated assembly. These connections are a revealing example of how hardware design decisions form a complex web of knock-on effects and compromises. The rationale behind these decisions will be briefly outlined in this section.

#### 2.3.1 Mechanical connections

A simple and reliable method of mechanically connecting the organ modules to the robot skeleton was required, one that would be suitable for autonomous assembly and also solid enough to not disengage or break when the robot is functioning. It also needed to be quick and easy to reverse so that the organs could be re-used in subsequent robots.

**FIGURE 5:**
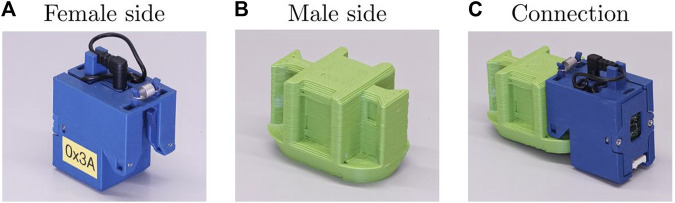
This figure shows the mechanical connector used in the ARE platform. The female side **(A)** uses two sprung arms to engage with the indentations on the male side **(B)** to form the final connection **(C)**.

It was therefore decided to use mechanical clips, shown in Figure 5, allowing the robot to be assembled simply by pushing the clips into place. The more complex female half of the clip is integrated into the organ casings where there is much more design freedom, enabling the male half to be simple enough in structure to directly incorporate into the printed skeleton.

#### 2.3.2 Electrical connections

All of the organs needed a means of being supplied with sufficient power, and to have a reliable communications link with the robot head. The chosen method of achieving this also needed to be suitable for autonomous assembly.

One approach could have been the use of independently powered, wireless organs. However, the practicalities of this were considered prohibitive, since each organ would have been much bulkier, heavier, more expensive, and complex to configure. Instead, a cabling system was chosen for this.

**FIGURE 6:**
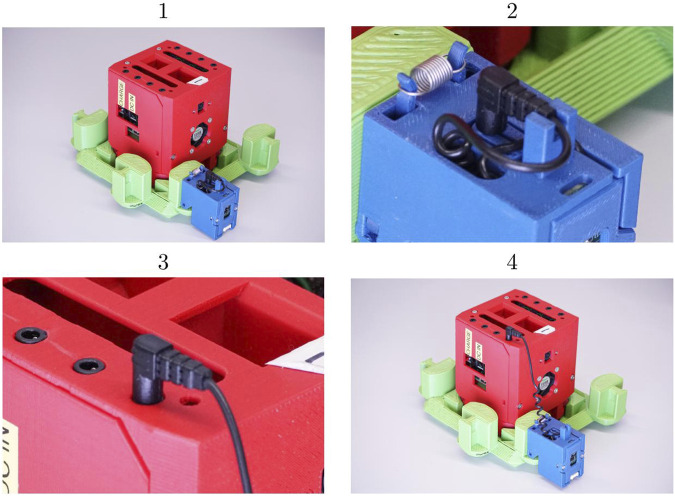
Retractable cables are used to interconnect organs, shown here at the different stages of the connection process: 1) The organ is clipped onto the skeleton. 2) The retractable cable is drawn out from a cavity in the organ casing. 3) The cable is connected to the head. 4) The coiled cable self-adjusts its length to the distance between the head and the organ.

For autonomous manufacture, it is advantageous for the chosen cabling to be easy to reliably insert, and tolerant of some misalignment. These attributes were obtained by using 3.5 mm TRRS (tip-ring-ring-sleeve) jack connectors, i.e., standard headphone cables in the four-conductor format sometimes used for hands-free headsets. They have the key advantages of rotational symmetry around the connector axis, and a tapered tip shape which can assist in compensating for misalignment during automated insertion. Coiled cables are used to avoid trailing lengths of excess wire, as shown in Figure 6.

### 2.4 Electronic hardware

Now a system has been established that can bring the building blocks of a robot together to realise novel body plans in hardware. However, the inner workings of those building blocks have not yet been considered. Robots cannot function without electronics, so the electronic hardware underpinning the platform is absolutely fundamental to its functionality. This is easily overlooked in simulation, where no electronics are required, and we may make the assumption that we can combine building blocks at will and expect them to perform more or less consistently in all configurations, but this is not true in reality.

The paramount aim here is reliability, because if a robot is subject to even intermittent electronic failure, it is effectively non-functional and cannot be evaluated. It is essential for the hardware to perform consistently under variable conditions, and for the limits of reliability to be clearly defined. Any robot configuration which exceeds those limits must be considered invalid and excluded from the *viable phenotype space*. Ensuring reliability of the electronic system primarily depends on the communications and power delivery infrastructure, and it is these areas that will form the focus of the remaining design description.

These challenges will be explored in greater depth, since they are critical to understand for implementing evolved robots in hardware, but rarely considered in the evolutionary robotics field. The remainder of this section will be structured as follows. Firstly, a brief overview of the electronic system used in the ARE platform, followed by a look at the communications infrastructure. Then, a detailed exploration of the crucial issue of electrical power, identifying the key challenges, followed by some examples of how they are addressed in the ARE electronics design. Finally, an example of how actuators can be adapted to improve the range of available phenotypes within the power constraints.

#### 2.4.1 Overall structure

**FIGURE 7:**
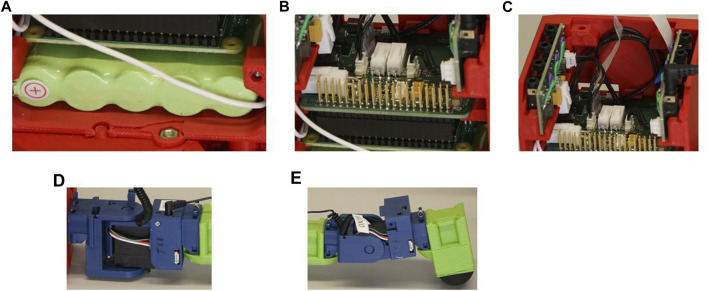
Components of the electronic hardware. The battery **(A)** is connected to a motherboard that is connected to the Raspberry PI **(B)**. To the motherboard is also connected a pair of daughter boards, each of which has four TRRS sockets for organ connections **(C)**. An example of daisy-chain topology with two joint organs can be seen across **(C–E)**, whereby a cable from the distal joint in **(E)** is plugged into a second socket on the proximal joint in **(D)**, which in turn is plugged into one of the organ sockets on the daughter board in **(C)**.

The electronic hardware comprises a set of bespoke printed circuit boards (PCBs), which together form a modular platform that can be arranged into different configurations to provide functionality for evolved robot body plans. The structure is shown in Figure 7. In the head organ is located the main motherboard PCB, which interfaces directly with a Raspberry Pi microcontroller (Figure 7B). This provides power regulation for the Raspberry Pi, an assortment of utility functions, and breakout headers for the communications bus and power. To these are connected two daughter board PCBs, each of which has four TRRS sockets to which organs can be connected with their cables, forming a star topology for the delivery of power and communications (Figure 7C). In the special case of the joint organ, a daisy-chain topology can be used, whereby the proximal joint connects to the daughter board as normal, but the distal joint can then be connected to a second socket on the proximal joint, allowing it to share the same power supply and communication bus segment (Figures 7D, E). In principle this can be used to form chains of any length, but in practice this is limited by the power system. This will be further discussed in later sections, after first describing the communication system.

#### 2.4.2 Communications

For the robot to control its sensors and actuators, some form of communication method is required. In a modular application, it is desirable to have a bus structure, in which devices can be easily added and individually addressed. I2C was selected for this, since it is compatible with a wide range of hardware and requires only two signal lines, enabling our single four-conductor TRRS cable to carry both communications and power for each organ.

I2C communication can fail if the total bus capacitance exceeds 400 pF, so a method is needed to limit the effect of the distributed capacitance in the cables, since this can vary arbitrarily between different robot configurations. Each TRRS socket on the daughter boards is equipped with a PCA9517ATP I2C repeater chip, to electronically subdivide the bus and prevent the capacitance from reaching a problematic level.

#### 2.4.3 Power is everything

The challenge of reliable power delivery turned out to be so fundamental to the design of the electronics for an evolvable robot platform that it merits a detailed explanation here.

At the simplest level, it should be considered that the power delivery capacity of any real system is limited. The rate at which energy can be drawn from a battery is not infinite, and electronic components will overheat and cut out if they exceed their rated current-carrying capacity. From this, we might imagine that this could be considered a simple scalar constraint on the maximum continuous current that can be drawn from the power system. Indeed, this limit must be adhered to, and it places restrictions on the number of actuators that can be used and their power. However, this is insufficient. A robot platform could be designed and operated within such a limit and still not function reliably. The principal reason for this is the effect of *load-dependent fluctuations in the supply voltage*, which can result in both inconsistent behaviour of sensors/actuators, and intermittent failure of microcontrollers.

**FIGURE 8:**
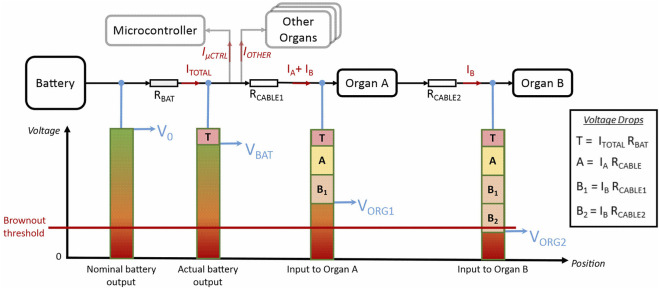
An illustration of how load-induced voltage drops manifest at different points within the power distribution network, using the example of a daisy chain comprising two organs. The total combined current draw of all components *I*_*TOTAL*_ induces a drop *T* in the battery output voltage due to its internal resistance *R*_*BAT*_. The current drawn by each organ then induces additional voltage drops *A* and *B* across the resistance of the cables, such that the supply voltage at the organ inputs is further reduced. Note that the current to Organ B must travel through both the first and second cables, so its effect is multiplied by its daisy chain position. In real operation the blocks in the diagram would dynamically expand and contract as the load varies, but when power budgeting we must allow for the worst case at peak current. If the sum of these drops at any point in the system brings the voltage below the brownout threshold, the robot will malfunction, so avoiding this is a necessary condition for reliability.

To understand this requires only Ohm’s Law *V* = *IR*, and an understanding of the non-zero electrical resistance between the power source and the active components of the system. As current *I* is drawn from the battery to power one of these components, it must pass through some resistance *R* on its way. Ohm’s Law tells us that this produces a voltage difference *V* across that resistance, hence a *voltage drop* will be observed—the local voltage at the component will be *V* lower than the source. The magnitude of this voltage drop is the product of the *current draw* *I* of that component, and the total *resistance* *R* through which that current must flow to reach it. These are key terms which will be used throughout the following explanation, with reference to Figure 8.

**FIGURE 9:**
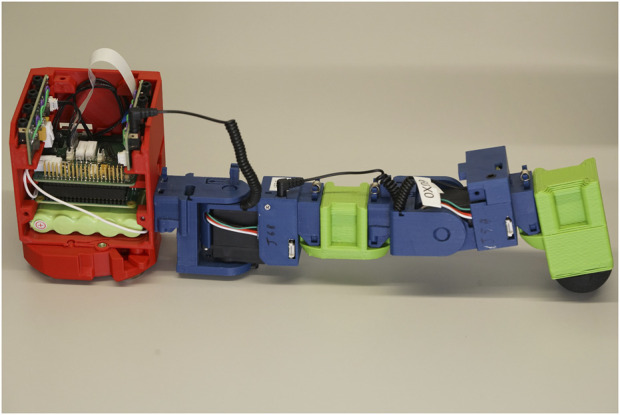
The physical equivalent of the example in Figure 8 with two joint organs in a daisy chain and other organs omitted for clarity. Current for the first joint flows from the green battery at the bottom of the head organ, up via the motherboard and daughter board and along the first organ cable. The current for the second joint follows the same path before continuing along the second organ cable, so its current must flow through both the first and second organ cables, increasing the impact of the second joint on the power budget.

Figure 8 illustrates how this phenomenon manifests in a robot with a chain of two joint organs as shown in Figure 9, whereby different voltage drops appear at each node of the circuit. These effects are highly interdependent, with all system elements contributing to a voltage drop at the battery, and each successive organ in a daisy-chain having a cumulative, non-linear impact on the voltage drops across each connecting cable.

This voltage variation is also highly dynamic. When actuators are under load, their continuous current draw increases in proportion to the torque they are exerting, and this increase may be 5–10 times greater than their no-load current. Imagine the forces in each joint of a limbed robot as it crawls around, or the motors of a wheeled robot colliding with obstacles, and this can give some idea of how much this load will fluctuate in operation. Furthermore, when an actuator accelerates, particularly on startup or direction reversal, it draws a transient spike of current well in excess of its normal maximum continuous current draw. Considering this in the context of the load-dependent voltage drop effects just described, it is clear that the voltages throughout the system will be subject to considerable fluctuations during operation, and this will depend on both the structure and behaviour of each evolved robot.

Why are these voltage fluctuations important? One reason is that the behaviour of motors, and certain sensors, depends directly on their supply voltage, so load-dependent variations in their behaviour will occur if this is not controlled, leading to divergence from the behaviour expected when evolving in simulation. Although not the focus of this discussion, it is worth noting that this is a complex reality gap issue that would be difficult to accurately model in simulation.

The second, and far more critical reason, is that digital electronics such as microcontrollers require a minimum supply voltage to operate, below which they will stop functioning. This condition is known as *brownout*, and if the load-dependent fluctuations are too great, the voltage will drop below this brownout threshold. Microcontrollers are functionally essential to a robot, and even a momentary brownout will cause them to reset, leading to malfunction or failure of the robot during operation. This means that *the prevention of brownouts is an essential condition for reliability.*

In a platform for implementing evolved robots, where the load conditions can vary arbitrarily between different evolved phenotypes, the point at which brownout occurs represents a limiting factor on how the robots can be configured, and phenotypes that exceed this limit must be excluded from the *viable phenotype space*. In other words, the range of feasible robot body plans available to evolution has a direct dependence on the ability of the power system to handle these load-dependent effects and guarantee a sufficient supply voltage to all the digital electronics on the robot.

To summarise, reliable and consistent power delivery is fundamental to a hardware platform for evolving robots, and achieving this requires a number of load-dependent factors to be considered, as summarised in Table 1. Excessive current may lead to damage or thermal cutout of electronic components, but a more complex challenge is presented by the dynamic voltage fluctuations produced as current is drawn through the resistances along the power distribution path to each organ. These can not only change the behaviour of the sensors and actuators on the robot, but will lead to malfunction if the supply voltage to any of the microcontrollers drops below its brownout threshold. Since the load conditions of the system depend on the specific configuration of each evolved body plan, it is not possible to design a system which can accommodate all possibilities—the scope of the exploration space is limited directly by the capabilities of the power system.

**TABLE 1:** The constraints imposed by the power system on the capabilities of the evolvable robot platform.

Constraint	Cause	Effect
Total current draw	Battery voltage drop due to internal resistance	Central brownout
Local current draw	Component overheating	Thermal cutout or damage
Organ-specific current draw	Voltage drop due to cable resistance	Peripheral brownout
Organ position in daisy chain	Compound voltage drops due to multiple cables	Peripheral brownout

“Central brownout” refers to a voltage drop at the battery output sufficient to brown-out the entire system, including the central microcontroller, causing complete robot failure. “Peripheral brownout” refers to a voltage drop at an organ input sufficient to brown-out its local microcontroller, causing that organ to fail.

It is in this sense that *power is everything* for evolvable robot design—the system as a whole can only ever be as capable as the limits of its power system. The evolutionary process that generates the robot body plans must operate within these limitations, and they must therefore be mitigated as much as possible through careful hardware design. Neither the evolutionary designer nor the hardware designer can afford to ignore them.

It is of note that the power issues described above were not well-understood at the start of the ARE hardware development, which meant that key opportunities to mitigate them were missed in early design decisions. This makes it a suboptimal example of careful, well-informed design as advocated in this paper, but a particularly interesting case study in coping with power constraints in a hardware system for evolving robots, since the constraints were more restrictive.

#### 2.4.4 Power distribution infrastructure

The power source in the ARE head organ is a 5-cell, 2Ah nickel-metal hydride (Ni-MH) battery, with a nominal output voltage of 6 V. This was chosen over lithium-polymer (LiPo) technology because the prevailing ambition at the time was a fully autonomous system where robots could run completely unattended and perhaps even be self-charging in the arena. The more complex charging requirements and safety implications of LiPo batteries would have made this prohibitively challenging, and a 6 V Ni-MH battery was the best available alternative that would fit within the desired form factor. This is a good example of how designing for ambitious goals can end up being detrimental to achieving realistic ones, because this battery choice was very limiting, as will be discussed later.

One consequence of this choice is that the nominal voltage of 6 V leaves little headroom above the 5 V operating voltage of the circuitry, and Ni-MH technology has a relatively high internal resistance. It was therefore a certainty that a robot under load would induce some voltage drops of sufficient magnitude to cause central brownout. To combat this, TPS63070 boost-buck voltage regulators were used. These can either step-down the supply voltage when it is over 5 V, or draw additional current to boost it to 5 V if the supply voltage is too low, thereby providing a stabilised output.

The Raspberry Pi is powered by a dedicated boost-buck regulator on the motherboard to protect it from fluctuations caused by the organs, and each daughter board features two further regulators, each powering two organ sockets. The daughter board regulators are configured to boost the organ supply voltage to 7.2 V, with each organ stepping this back down to 5 V with a smaller local buck regulator. The aim of this is to better ensure a consistent supply voltage, which would otherwise be subject to fluctuations due to the cable resistance.

Transmitting power at a higher voltage also allows lower transmission current, which reduces the impact of the cable resistance—a similar principle to that used in high-voltage overhead transmission lines. A higher boost ratio such as 12 or 24 V would likely have been preferable, but this was not fully understood at the time of selecting 7.2 V, and this choice was made somewhat naively to allow what was believed to be adequate headroom without the added complexity of a very high boost ratio, which would have required higher-rated components and made the regulators more susceptible to overheating.

Having explored the power and communications infrastructure, the final subsection describes some specific adaptations to the joint organ electronics to make the most of the limited power budget available.

#### 2.4.5 Power budgeting with servos

The joint organs for the ARE project are actuated by servos, which are of particular interest in an evolutionary robotics context because they can be used to produce limbs and evolve novel locomotion behaviours. Unfortunately, they are also particularly demanding devices from an electronics perspective, as they draw significant continuous current under load, and large peak transient currents when they initiate movement. This is further complicated by the need to daisy-chain multiple joints together to form limbs, which causes compound increases in the voltage drops in the cables. This means that servos represent by far the greatest challenge when it comes to power budgeting, and provide an instructive example of how hardware can be adapted to improve the scope of the *viable phenotype space*.

Recall that the reliability requirements of avoiding brownout impose a power budget in which both the current draw and daisy-chain position of an organ determines how much space it must be allocated in that budget. This restricts both the total number of servos per robot and the length of individual limbs, reducing the range of allowable body plans. In the case of our particularly limited power system, it would have made most limb configurations unworkable. How can we improve this situation and broaden the exploration space for evolution? The solution is to control the impact of servos on the power budget by limiting their current draw.

How much space does each servo really need in the budget? Space must be allocated according to the worst-case scenario in which all servos are drawing their maximum current, because otherwise there is a risk of brownout occurring when they peak simultaneously (such as at startup). However, this is wasteful, because the peak transient current is significantly greater than the actual continuous current requirement under load, so most of the time this would leave unused capacity in the system.

Furthermore, not all joints require the full output power of the servo—some may require maximum torque to lift the full weight of the robot with a long moment arm, but others may only need to exert smaller forces, for example, sweeping limbs forward and back during locomotion. Imagine a human with a quadriceps muscle for every muscle in their body. This would be a highly inefficient design, but in effect this is the only type of arrangement available to evolution when all the servos are identical and unconstrained—all the “muscles” have the same power, whether they need it or not.

These two observations indicate that we could increase the range of possible configurations by selectively limiting the current drawn by each servo, thereby reducing their power budget allocation to only the amount that is needed. The same principle can also be applied to other actuators, such as the DC motors found in the wheel organs. To achieve this, a programmable current limiting circuit was implemented.

The circuit uses a MAX17613AATP+ current limiting chip, whose current limit can be set with a resistor. By using a programmable resistor combined with an appropriate series resistor, we can make the current limit programmable within a defined range. In the case of the joint organ, an AD5246BKSZ10-RL7 digital potentiometer in series with a 3.6 K resistor allows the current limit to be programmed over I2C between about 330 and 1250 mA. The current limiter works by dynamically reducing the voltage supplied to the servo when the measured current draw reaches the limit, thereby preventing the current from increasing any further.

At the upper limits, this can be used just to control the large peak transients without any loss of holding torque, but the current limit can be further reduced to restrict the maximum torque available to the servo in exchange for more space in the power budget—effectively we can trade off having some weaker “muscles” in order to have more “muscles” in total.

The only requirement for this is that the actuator is able to tolerate these variations in supply voltage. For a normal DC motor, as in the wheel organ, this is no problem at all—the motor simply has a reduced maximum torque and accelerates more slowly. Servos, by contrast, have a threshold below which they stop behaving correctly, due to their internal electronics.

Hobbyist-type servos come in two variants, analogue and digital, and this current limiting technique was first tested with the popular Towerpro MG996R, which is a digital servo. However, even very modest current limiting caused it to lock up in a kind of twitching paralysis. This is because it contains digital electronics that brown-out when the current limiter drops the supply voltage, and this happens repeatedly due to the high startup transient of the servo, inducing a reset loop. Analogue servos, by contrast, do not use digital control circuitry and are much more tolerant of undervoltage, so the FEETECH FS5115M-FB servos selected for the joint organs enable the “muscle strength” (maximum torque) to be controlled by the current limiter as desired. At the lowest current limits, these servos do exhibit a “struggling” behaviour if the load is too great, in which they repeatedly attempt the same movement rather than just holding a reduced torque, but this is a more graceful and organic behaviour than total paralysis.

To summarise this section, the use of programmable current limiters in combination with analogue servos (or DC motors) enables actuators to be implemented in which their occupancy of the power budget can be selectively reduced to only the amount that is needed, from simply controlling excess transients to limiting their “muscle power” This thereby expands the range of available robot configurations in the *viable phenotype space*, without any increase in the power supply.

This concludes the description of the hardware design, and Section 3 will now explore the interaction of this hardware with the evolutionary process.

## 3 Results

### 3.1 Overview

The most striking finding of the ARE project hardware design was the profound impact of hardware design decisions on the regions of the evolutionary space that could be reached, revealing the necessity of integrating an in-depth understanding of evolution into the hardware design, and an equally thorough understanding of the hardware into the evolutionary design.

Both the mechanical and the electronic hardware imposed constraints on the space of feasible phenotypes, and these constraints will be examined in Section 3.2, before exploring in Section 3.3 the impact of those constraints on the evolutionary process.

### 3.2 Hardware constraints on evolution

#### 3.2.1 Manufacturable morphologies

Recall that the ARE system produces robots by 3D printing an evolved skeleton, which integrates a standard base into which the head organ is inserted. This then forms a central grasping point for the remaining assembly, whereby organ modules are clipped onto printed mating points on the skeleton, and their connecting cables are plugged into the sockets at the top of the head organ. In principle, this seems like it should provide for a very rich morphological space of robot body plans that can be evolved. In reality, however, it was more limited than first envisaged, and this is because of decisions made when choosing how the robots would be manufactured. Any production method will create constraints in the evolutionary space, and the choices made in this area will directly determine both the placement of those constraints and the magnitude of their influence.

The first constraint is simply, scale. Although the 3D printers have a relatively large build plate for printers of their class (280 mm × 280 mm), a substantial portion of this is taken up by the standard head organ base, measuring approximately 130 mm square. This leaves a relatively small workspace for evolution to develop interesting morphological structures—a margin around 75 mm wide. This is compounded by the large feature size imposed by the oversize extrusion nozzle and the bulky organs, which must be large enough to accommodate circuit boards, cable storage and mechanical clips in addition to the sensors and actuators themselves. Each mounting point printed onto the skeleton occupies 20 mm × 38 mm in the build plate area, and the skeleton generation algorithm is based around an 18 mm voxel size, so it is clear that there is limited room for structural variation within that 75 mm margin.

There is more space in the vertical direction within the 250 mm high build volume, but the need for the robots to be physically assembled creates no-go areas within this space too. The organ clips slide onto their mounting points from above, so each mounting point requires an area of free space above it. Similarly, the area above the head organ must be clear of other structures to allow space for the robot arm to insert it into the skeleton, and then insert the cables into it from above.

Visualising the remaining space in which structures can be built, this leaves a *tall square torus-shaped volume with voids wherever there is an organ clip*, a much more limited space for morphological variation than one might think when imagining the possibilities of 3D printed robots.

The limitations of FDM printing itself impose more complex constraints. Overhanging structures forming an angle shallower than 45° to the horizontal require additional support scaffolds to be printed underneath them, and this extra material must be manually removed later. The fully automated manufacture in ARE could not handle this kind of post-production work, so overhangs had to be either avoided entirely or algorithmically modified to integrate sloping structures underneath—a major restriction on the shapes that could be built.

More significant, and perhaps less obvious, is the building-up of the model from a flat build plate. This means that one side of the robot must always be completely flat, and since in this case the head organ is inserted from the top in a vertical orientation, this flat side must be the underside of the robot. There can be no organ clips on the underside, no printed structures may extend below the bottom edges of the robot, and the underside will be completely smooth without morphological features.

Considering how a ground-based robot interacts with its environment, the structure of its underside is of particular importance, because it determines the ground clearance of the body and how any wheels and limbs will engage with the terrain. The evolutionary process could also generate morphological structures in the skeleton to assist in overcoming obstacles or uneven surfaces if it were free to operate on the underside. In this way, the imposition of a flat planar boundary here is more restrictive than a simple limit on the morphological space—it prevents the evolutionary process from accessing one of the most useful areas of variation.

One approach to address this might have been to print the skeleton in a sideways or inverted orientation, and rotate it at assembly time, but the head organ attachment method prevents this, as it relies on a vertical cavity that is open at the top. This dependency in turn arises from the requirements of the robot arm assembly system, the design of the organ interconnections, and the need to avoid printed support material, such that implementing this change would require major overhaul of multiple system elements. This illustrates once again the deeply interlinked nature of hardware constraints.

An important conclusion of these observations is that the morphological space is drastically altered by the requirements of manufacturability. Every decision made about how the robots will be constructed has an influence on how this space is constrained. These constraints can be made more favourable by careful choice of implementation details, but they cannot be avoided, and this means that practical robot evolution is inherently dependent on the realities of the chosen production method.

#### 3.2.2 The Tyranny of power

Although trivially easy to ignore in simulation, electrical power is one of the most fundamental limiting factors in an evolvable robot system. All active components of a robot require power to operate, and if the power supply cannot meet the demand, parts of the robot will malfunction or stop working altogether. This defines a “power budget” within which evolution must operate when adding active components to a robot body plan.

As outlined in Section 2.4.3, the power budget is not so much a fixed figure as a set of rules that constrain the total number of organs, where they can be placed, and how much power each one can consume. Table 1 summarised these principles and how they relate to three failure modes in the power system: 1) central brownout, 2) thermal cutout and 3) peripheral brownout. The specific power budget imposed by the ARE hardware may be illustrated in terms of how the system must be constrained to avoid each of these failure modes.

**FIGURE 10:**
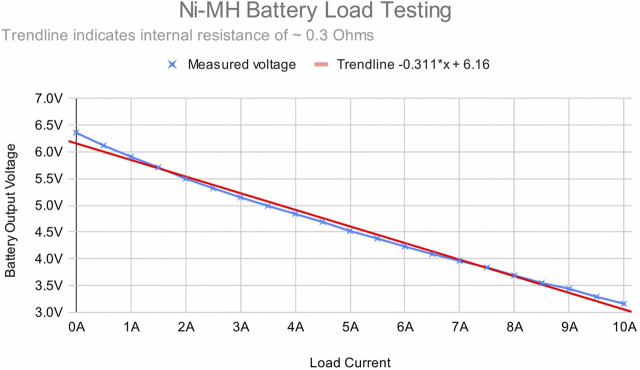
The output voltage measured across the terminals of the 5-cell Ni-MH battery at different load currents. The gradient of the current-voltage line indicates the internal resistance of the battery, approximately 0.3 Ω.

*Central brownout* is battery-dependent and defines the total allowable system current. Figure 10 indicates that the battery output drops below the operating voltage of 5 V for loads exceeding around 3.5 A. For context, this would accommodate only two joint servos at their maximum 1.25 A current limit, if allowing 1 A for the microcontroller and internal components. Robots with sensors and current-limited wheel organs could be built, but current limiting would be essential, and most limbs would be unachievable.

**FIGURE 11:**
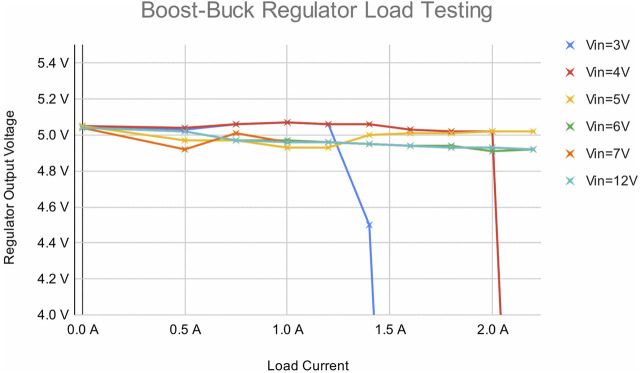
The output voltage measured at the output of the boost-buck regulator circuit at different load currents and input voltages, showing that it can easily step down from higher voltages, but the extra switch current required in boost mode limits how low the input voltage can go. Sudden drops indicate thermal cutout of the regulator, showing the limits of its output capability.

By powering the central microcontroller from a dedicated boost-buck regulator, the limiting factor for central brownout becomes the point at which the regulator overheats and cuts out. This is harder to define precisely, but it can be inferred from Figure 11 that this occurs somewhere around 3 V, granting an extra 2 V of headroom. Returning to Figure 10, this new 3 V threshold allows for a total load up to around 10 A, and a significantly broader range of robot configurations.

*Thermal cutout* determines the limit on the local current draw from the organ sockets. Each pair of sockets is powered by a regulator with a nominal max output of 2 A, so this limit must be divided between each pair. A single organ chain may draw up to 2 A, but the load on the neighbouring organ socket must be reduced accordingly. Due to the size and weight of the robots, a sufficiently powerful two-jointed limb for load-bearing requires more than 1 A, so although there are eight sockets, in practical terms the system can support a maximum of four limbs for locomotion.

*Peripheral brownout* is the most complex effect to calculate, and limits the allowable limb configurations. The TRRS cables have an unusually high resistance of around 1 Ω, meaning that for every 1 A of current, they induce a voltage drop of 1 V per cable. The daughter board regulators use a transmission voltage of 7.2 V, which allows 2 V of headroom. Table 2 shows some examples of allowable two-joint configurations using the available range of current limiting. Notice the effect of daisy-chain position on Joint 2; additional power here has a greater cost. It can never use the full limit of 1.25 A, unlike Joint 1, and the power available to the limb as a whole is reduced as the limit for Joint 2 increases.

**TABLE 2:** Some examples of allowable two-joint limb configurations within a 2 V voltage drop limit, using the available range of current limiting.

Joint 1 limit (A)	Joint 2 limit (A)	Cable 1 current (A)	Cable 2 current (A)	Total Vdrop (V)
1.25	0.37	1.62	0.37	1.99
1.0	0.5	1.5	0.5	2
0.5	0.75	1.25	0.75	2
0.33	0.83	1.16	0.83	1.99

Joint 2 is the second in the daisy chain, so its current must flow through both cables, producing a greater impact on the power budget. This means it has to use lower current limits than Joint 1 for equivalent pairings, as highlighted in blue.

To conclude, the power system imposes complex, interdependent constraints on allowable robot configurations. Choosing higher-performance components can expand these constraints, but other practical considerations may limit these choices, and even a very powerful system cannot match the unconstrained power assumed by simulation. Any system for evolving real robots will have to account for a finite power budget in its design.

### 3.3 Effects on evolution

The ultimate goal of the Autonomous Robot Evolution project is to integrate two or more evolutionary processes, including a single process in simulation and a single process in hardware to create robots adapted for challenging environments. Specific details of the ARE evolutionary processes are outside the scope of this paper, so the following discussion is limited to qualitative observations. However, further information may be found in (Hale et al., 2019; Le Goff et al., 2022).

This section presents a reflection on the direct and indirect influence of the hardware constraints on the generation of robot body plans by the evolutionary process, before addressing the specific challenge of avoiding unfeasible phenotypes.

#### 3.3.1 Direct hardware influence—body plan boundaries

The direct hardware influence refers to the effect of the fixed constraints imposed by the hardware implementation which are directly incorporated as limits in the evolutionary process.

The star topology described in Section 2.4.1 defines a maximum number of 8 organs that can be connected directly to the head. Although this limit is greater than other platforms in literature (Jelisavcic et al., 2017; Auerbach et al., 2018; Miras et al., 2020), the genome decoding has to accommodate this limit.

The limitations of power budgeting described in Section 2.4.3 restrict the allowable number of joints and how they may be configured. The daisy chain length limit of 2 joints is one example, meaning that only simple limbs with 2 degrees of freedom can be implemented with this system, and this limit has to be defined in the decoding. Therefore, the decoding sets a limit on the number of joints that can be daisy-chained together. It is not possible to build robots that require a longer chain of interconnected joints such as the snake-like morphologies evolved in (Miras et al., 2020).

As described in Section 3.2.2, limbs are configured by allocating an appropriate power limit to each joint, and this can be done in a limited variety of ways. This is important as it could drive the evolutionary process in different directions. For example, if high power is allocated to proximal joints and low power is allocated to distal joints, then crawling behaviours might be seen in the robot. Robots with this approach might make more use of caster balls to move. On the other hand, if low power is allocated to proximal joints and high power is allocated to distal joints, then more behaviours of the robot lifting itself may be seen. It might be interesting to explore this domain further by allocating different proportions of power and analysing the different behaviour in the robots and their influence on the evolution of the body plans.

#### 3.3.2 Indirect hardware influence—the curse of the ring-shaped robots

The indirect hardware influence refers to emergent effects observed in the evolved body plans, which result from the hardware design decisions. In this section, an example is presented, following the process from genome decoding to the types of robots produced.

Each body-plan is encoded indirectly by a compositional pattern-producing network (CPPN) (Stanley, 2007). When decoding, the coordinates of a 3D matrix are used to query the CPPN, which returns values indicating whether a voxel of skeleton material should be placed at a location. After all positions are queried, a repair function ensures that the skeleton is printable, e.g., removing disconnected plastic and/or overhangs. Additional outputs indicate whether (and where) organs are attached to the skeleton.

One of the rules set in genome decoding is that, regardless of the evolved morphology specified by the genome, every skeleton must include a ring-shaped base around the head organ. This decision was taken to ensure that the evolutionary process would always have somewhere to place organs on the skeleton, since unviable robots with no organs could otherwise be generated. However, this rule when combined with the 3D printer build plate constraints described in Section 3.2.1 created an undesired outcome in the resulting process.

**FIGURE 12:**
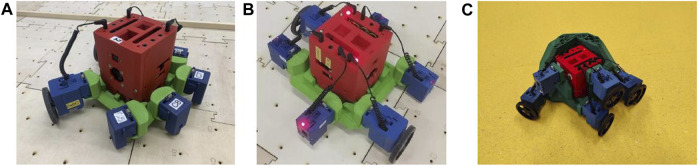
Example of evolved robots. **(A)** This robot has 2 wheels on one side of the robot and 4 caster balls on the other side and this robot is capable of moving in a straight line by the caster balls getting stuck between the gaps of the tiles. **(B)** This robot has two wheels, one on each side, and four sensors. This robot has a tendency of leaning towards either side. The best behaviour can be seen if the robot leans towards the left side, however, if the robot is leaning on the right side, the robot reverts to the left side by hitting the gaps between the tiles. **(C)** This robot uses the bulk plastic to nudge its way around obstacles instead of avoiding them.

The evolutionary process has a tendency to produce a high number of planar, ring-shaped robots (Figures 12A, B), displaying only limited variation around the base of the skeleton. Although it has the option of generating structures higher up, the usefulness of placing organs and skeleton features there is limited in a ground-based arena, and the confined 3D printer build space prevented the development of more elaborate features at floor level. This is a clear example of how hardware constraints define the range of robots that can be evolved.

It is important to highlight that the way the genome is decoded by having the rule of the ring-shaped base contributed to the abundance of these types of robots. The evolutionary process settings could potentially be adjusted to ameliorate this constraint. One or more of the following could be changed: 1) genome decoding, 2) task and 3) fitness evaluation.

Despite this limitation, for some rare examples, the evolutionary process was able to generate novel skeletons with a functional benefit. For instance, the robot shown in Figure 12C has a wedge-shaped structure that enables it to navigate around obstacle corners without needing to sense or avoid them. This example hints at the potential for morphological evolution to exploit this technology to produce interesting adaptations, but the constraints on the morphological space were such that it could only use a small fraction of this potential.

To conclude, hardware constraints can drive the evolutionary process to create robots with similar features, particularly when the varying degrees of freedom permitted by the hardware implementation are poorly matched to the types of variation most useful for adapting to a particular task. This lack of diversity may be addressed to some extent by optimising the hardware design, but where this is impossible, the evolutionary process (task, fitness function or genome decoding) needs to be adjusted to work around these limitations. Therefore, it is highly important to consider these factors during hardware design and when designing the evolutionary process.

#### 3.3.3 Enforcing feasibility

The hardware constraints mean that many of the robots that can be defined by the genome representation are not practically feasible, so it is desirable to find ways of making the evolutionary process generate only robots that can be implemented. Two methods were used to achieve this: *phenotype filtering* [also known as *genotype filtering* in Eiben (2021)] or *phenotype repair*.

The *phenotype filtering* method consists of discarding all robots that are not feasible from the population. This is achieved by assigning them the lowest fitness score and removing the probability of these robots being selected for the next-generation. However, as shown in Hale et al. (2019), Buchanan et al. (2020a) (illustrated in Figure 1), large proportions of evolved robots would get discarded by this filtering step, reducing the diversity in the population. In other words, genetic lineages keep getting cut off by unfeasible robots along the way, making it difficult for evolution to traverse the fitness landscape, which then allows a limited number of remaining lineages to overtake the population. Kriegman et al. (2020a) applied a similar phenotype filtering to evolved biological organisms but with the main difference that the filter was applied to the final set of organisms produced. A similar loss of diversity occurs with this method.

The *phenotype repair* method consists of applying changes directly to the decoded phenotype to make it feasible (Hale et al., 2019; Buchanan et al., 2020b). The diversity of robots increases and the landscape becomes easier to traverse as the lineages are not getting cut off and the diversity of robots does not decrease as much as with *phenotype filtering*. However, because the repair modifies the phenotype after decoding, this method increases the distance between the genotype and the phenotype, such that small changes at the genotype level could produce either no change at all or very big changes at the phenotype level. This is shown in Le Goff et al. (2022) where many of the robots share similar features to each other. This becomes a problem when a good robot (but not optimal) is found and smaller changes are required to improve it.

In addition to these two methods, numerous other constraint-handling techniques exist in the evolutionary computation literature, many of which are summarised in Coello (2022). Although not specifically designed to cope with hardware constraints, it may be that some of these approaches could be applied to navigate the feasible regions of the phenotype space more effectively. However, all such methods, regardless of effectiveness, are limited to working with the *viable phenotype space* imposed by the implementation—they cannot change the space itself to make it more favourable.

An alternative possibility could be to design the genome to always decode into feasible robots without the need for methods like *phenotype filtering* or *phenotype repair*. One example can be found in chemistry, where Krenn et al. (2022) demonstrated that by changing their representation, valid molecular graphs could always be produced without any filtering. Brodbeck et al. (2015) encoded the building sequence directly into the genome to maximize the number of feasible robots, such that out of the total of 500 robots, 96% were feasible. The authors also highlighted the existing trade-off between robot complexity and feasibility, where the challenge of creating feasible robots increases with their complexity. For example, modular robot platforms like (Faina et al., 2015) can be encoded to inherently manufacturable with a tree-like representation, but the richness of the morphological space is greatly reduced.

In conclusion, both *phenotype filtering* and *phenotype repair* use a post-decoding step to restrict the evolutionary process to generating feasible robots, but both introduce their own disadvantages that make it harder for evolution to work effectively. It may be that other constraint-handling methods from evolutionary computation could improve upon this, but all such methods are compromises, limited to attempting to compensate for the challenges already imposed on the search space by the system design. Designing for inherent manufacturability in the genome is an alternative approach, but this restricts the richness of the morphological space. It is therefore desirable to reduce the need for such methods by considering the effects of the hardware implementation in all aspects of the evolutionary system design.

## 4 Discussion

In this paper, we have identified that the design of an evolvable robot platform in hardware presents an unusual design paradigm, in which a fixed functionality specification is not known ahead of time, and instead the hardware design comes first and determines the range of functionality available to evolution. Each decision made about how to manufacture the evolved bodies and connect their mechanical and electronic parts together influences the constraints on the range of shapes that can be constructed, and the limitations of the underlying electronics introduce further constraints on allowable body plans.

When evolving in simulation, these constraints are easily overlooked and rarely considered, but they are fundamental to the goal of building evolved robots in hardware. By exploring the example of the ARE framework, we have illustrated how such constraints can manifest in an evolutionary system, and how the design of both the hardware itself and the evolutionary processes can determine the nature of those constraints, as well as attempt to ameliorate their impact on the achievable diversity and usefulness of the evolved robot population. In doing this, we have highlighted the critical importance of this interplay between evolution and hardware. These two sides can be brought together and summarised using the concept of *viable phenotype space*.

### 4.1 Viable phenotype space

What is the *viable phenotype space*? At the beginning of this paper, we defined it as follows:

*“The* ***evolvable phenotype space*** *is defined as the complete set of possible phenotypes that could be generated by an evolutionary process within a particular genetic representation”*

*“The* ***viable phenotype space*** *is defined as the subset of evolvable phenotypes that can be implemented and reliably evaluated in hardware, after manufacturing constraints and hardware limitations are taken into account”*

**FIGURE 13:**
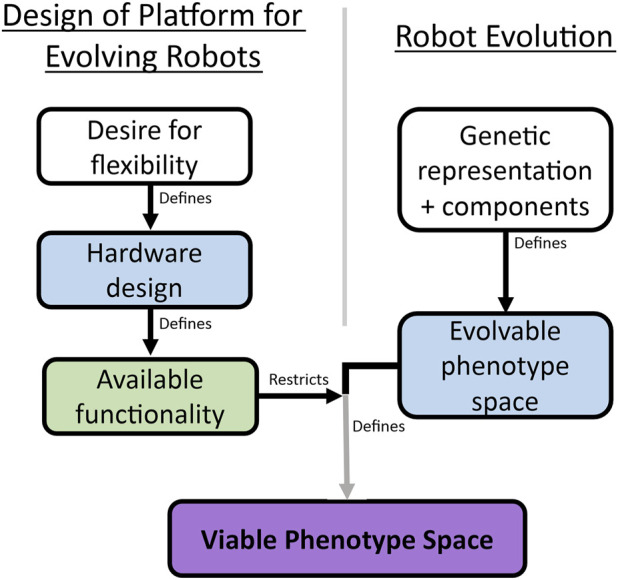
This diagram brings together the hardware design paradigm described in Figure 2 with the “design” space available to the evolutionary process. The hardware constraints define which regions of the complete *evolvable* space defined by the representation are practically feasible, and the combined result of this is a more restricted *viable phenotype space*.

The relationship between these two spaces and the evolutionary system design is illustrated in Figure 13, showing how both the engineering and the evolutionary algorithm aspects come together to define the *viable phenotype space*.

In the example of the ARE system, the *evolvable phenotype space* comprises arbitrary combinations of skeleton voxels anywhere within a matrix the size of the 3D printer build volume, any combination of different organs can be connected at any positions on the skeleton, and joints can be daisy-chained to build a limb of any length.

The *viable phenotype space*, by contrast, is constrained to structures within a narrow ring-shaped space around the head organ, in which there may be no overhangs beyond 45°, and no features below the flat plane of the underside, with the further restriction of an empty area above each organ to enable assembly. Electronic limitations confine the organs to a total of eight organ sockets drawing a maximum combined 8 A of current, with a complex power budget governing whether a given organ can be connected or daisy-chained at a particular position, depending on its individual power requirements and those of other organs in the system. This presents a much more convoluted and restrictive landscape for evolution.

What is the effect of these restrictions? The objective of the evolutionary process is to efficiently traverse the *evolvable phenotype space* in search of the best robot design, and it does so by generating variations in the genome and evaluating the effectiveness of the resulting phenotypes. However, since many of these variations will produce robots that cannot be implemented and evaluated in hardware, navigating the *viable phenotype space* presents a different challenge to that experienced in simulation—evolution is presented with additional obstacles. We make the following observations about these obstacles:

• Boundaries: Variation is only possible within a confined space. Any evolved phenotype which exceeds the limits of this space must be modified or discarded from the population, for example, if a structural change produces a non-manufacturable feature.
• Interdependence: These boundaries are not simple fixed limits, because every part of a robot has an effect on the other parts—a feature which is valid in one configuration may not be valid in another. The ARE power system is a clear example of this, where the effect of a particular actuator on the power budget depends not only on its power requirements, but also on its position within a daisy chain and the configuration of all the other organs on the robot.
• Fragmentation: The remaining feasible regions of the phenotype space are not contiguous, but spread out and broken up by many unfeasible regions. For any given genetic change, there is a chance it may result in a phenotype that cannot be implemented, preventing it from forming part of a developmental trajectory. This makes it more difficult for an evolutionary process to explore and exploit the space effectively.

It is clear from these observations that the task faced by the evolutionary process is deeply interwoven with the constraints introduced by the hardware implementation. Therefore, in the design of any evolutionary system which is intended to produce real robots, it is necessary to incorporate detailed consideration of this *viable phenotype space* from the outset.

On the evolutionary side, it is critical to consider the constraints of the hardware in order to navigate the exploration space effectively. An approach that is highly successful at exploring the *evolvable phenotype space* in a simulation environment may perform poorly when required to work within the *viable phenotype space* and generate robots that can be implemented in hardware. The real goal, therefore, is to identify which regions of the phenotype space defined by the genetic representation contain robots that can actually be implemented, and find a way to restrict the evolutionary process to operate effectively within those regions.

A possible approach to this is the application of post-decoding methods such as phenotype filtering or repair, but we have seen that this can have adverse effects on the evolutionary process, making it harder to generate diverse populations or evolve incremental refinements to individuals. An alternative approach is to design a genetic representation which is more inherently manufacturable, but this will have practical consequences for the flexibility of the resulting system, as only certain types of structures lend themselves to inherent manufacturability. Whatever the approach, the objective must be to optimise the evolutionary process to produce the best performance within the *viable phenotype space*.

On the engineering side, the design decisions made in the hardware implementation will to a large extent define the *viable phenotype space*, so careful consideration of this will lead to better choices about how the robots should be built. A completely unconstrained system like that found in simulation is not achievable, and the design of the ARE hardware has shown that “as flexible as possible” is also too broad a design goal, because at some point we are forced to choose which *type* of flexibility takes priority. Constraints inevitably have to be balanced against each other, and these choices should not be arbitrary if the system is to be truly effective. Instead, the aim should be to consciously make these choices with reference to the *viable phenotype space*, in order to make it as *useful* as possible. In practical terms, this means that the hardware design should aim to maximise the degrees of freedom in the phenotype space which are the most relevant to the problem at hand, and any obstacles to smooth variation within these degrees of freedom should be minimised.

### 4.2 Broader applicability

Having examined the specific example of the ARE system in detail, it may seem that the issues of the *viable phenotype space* are particular to this system, so let us consider these ideas in a broader context.

An interesting mechanical parallel to the problem of peripheral brownout may be found in the EMERGE system presented by Moreno and Faiña (2021). In this system, the mechanical connections are made magnetically, and these can become disconnected under excessive force. This limits the allowable torque that can be exerted by the actuators, and connections can become detached during evaluation. This is an example of an intermittent, load-dependent failure mode similar to brownout. Their work treated this as a fitness limitation whereby the travelled distance was reduced, but this arguably should be regarded as a binary reliability issue. In a practical application, it would not be allowable for robots to fail intermittently, and phenotypes with a high risk of violating the torque limits of the connections would have to be excluded from the population. Any practical system would be expected to require some limits of this nature on allowable structural or electrical load.

Brodbeck et al. (2015) examined the effect of manufacturing constraints on robot evolution using cubic modules autonomously glued together by a robot arm. They identified several manufacturing constraints that limited the structures that could be produced, and analysed their effect on diversity. They found that the diversity of the population was strongly restricted by these limitations, and removing one or more constraints led to an improvement in diversity, showing that there is a strong relationship between how the robots are constructed and the resulting ability of evolution to find novel solutions. Low diversity was also correlated with converging to local maxima, with populations being taken over by one type of morphology, highlighting why this issue is so important.

Some evidence of the advantages offered by the principles advocated in this paper can be found in the example of Faina et al. (2015). They present a heterogeneous modular system in which the *viable phenotype space* has been carefully considered in the hardware design process, analysing the kinematics of a range of possible tasks and using this as a basis for the motion primitives to be implemented as modules. They describe this as designing “evolution friendly” hardware, and are able to produce a range of functional robots as a result of taking this approach, demonstrating high diversity.

All of the above examples benefit from a discretised modular architecture, which lends itself well to inherent manufacturability, as they can be assembled blockwise in the manner of Lego. Indeed, all previous hardware work of this kind has used some form of branching structure, which greatly simplifies the phenotype space. The ARE framework, by contrast, uses a semi-modular system, where a free-form structural body is combined with modular organs. This is more susceptible to generating non-viable phenotypes, making the challenges of the *viable phenotype space* significantly greater than in related work. However, the semi-modular approach provides both a higher degree of flexibility and greater biological plausibility. For example, the taxonomic class Mammalia includes a vast range of body shapes and sizes, yet all mammals share the same organ designs, including vascular systems, digestive systems, etc., with remarkably little variation between species.

There is reason to believe, therefore, that as robot evolution gets closer to practical or scientific applications, there will be a greater need for the flexibility of a semi-modular approach. At the same time, requirements for manufacturability and reliability will necessarily become more stringent in order for such systems to be ready for real-world deployment. We are therefore confident that our observations regarding the *viable phenotype space* are likely to become increasingly important in future work.

## 5 Conclusion

The ultimate objective of the evolutionary robotics field is to evolve robots that are of practical use in real-world applications. To achieve this, it is necessary to progress beyond simulation and implement them in hardware, and address the challenges that this entails. It is well-known that there exists a reality gap between simulation and hardware, which leads to behavioural differences between virtual robots and their real counterparts, but this is not the only challenge. The realities of hardware implementation also have a profound effect on the evolutionary landscape itself, and the implications of this are far less explored.

In this paper, we have examined in detail the interplay between an evolutionary robotics process and the hardware with which the evolved robots are to be implemented. We have seen that *the evolutionary process is not separable from the hardware*, because the many constraints introduced by the hardware fundamentally define the nature of the phenotype space that the evolutionary process is to explore.

Because of this, *the hardware is also not separable from the evolutionary process*, because a conventional design approach cannot be applied to an undefined specification, and the objective instead becomes placing the hardware constraints in a way that maximises the useful design freedom available to evolution.

This work therefore identifies two key principles for future work in evolutionary robotics. One is that a hardware designer creating an evolvable robot platform must have an understanding of the evolutionary process and consider the effect of their decisions on the *viable phenotype space*. The other is that an evolutionary algorithm designer must have an awareness of how the constraints imposed by hardware change the nature of the exploration space for evolution, and consider how the evolutionary process may be optimised to exploit the feasible regions of that space more effectively.

## Data Availability

The raw data supporting the conclusion of this article will be made available by the authors, without undue reservation.

## Footnotes

1 Website: https://www.york.ac.uk/robot-lab/are/

2 An example of production can be found in this video https://youtu.be/cXChkZloPN4

3 Website: https://lulzbot.com/store/taz-6
